# Spatial barriers impact upon appropriate delivery of radiotherapy in breast cancer patients

**DOI:** 10.1002/cam4.1304

**Published:** 2018-01-22

**Authors:** Fabrizio Stracci, Fortunato Bianconi, Chiara Lupi, Manuela Margaritelli, Alessio Gili, Cynthia Aristei

**Affiliations:** ^1^ Department of Experimental Medicine Section of Public Health University of Perugia Perugia Italy; ^2^ Umbria Cancer Registry Perugia Italy; ^3^ Department of Surgery and Biomedical Sciences Section of Radiation Oncology University of Perugia and Perugia General Hospital Perugia Italy

**Keywords:** Breast cancer, public health, radiotherapy, spatial barriers to care, standard of care

## Abstract

Radiotherapy (RT) is the standard treatment for breast cancer patients after conserving surgery or mastectomy when patients are at high risk of relapse. Major obstacles to appropriate RT delivery are journey times. Since studies on access to RT were carried out mostly in large countries, this study investigated factors in an Italian region and the influence of RT delivery on survival. A total of 4735 female candidates for RT were included in the study. A geographic information system calculated journey times from patients' homes and surgery hospitals to RT centers. Logistic regression analyzed the influence of journey times, socioeconomic status, and other factors on RT delivery. Survival probabilities and excess mortality were assessed in 4364 propensity score‐matched patients. Journey times of 40 min or less from residence and from surgery hospital to RT center played a major role in access to RT. A large survival difference emerged between treated and untreated breast cancer patients. The excess mortality for untreated patients compared with propensity score‐matched women receiving RT was 3.1 (95% CI: 2.2–4.3). Expansion of RT facilities during the 11‐year study period improved RT delivery and outcomes by increasing availability but mainly by shortening journey times.

## Introduction

Radiotherapy (RT) is standard treatment after breast‐conserving surgery (BCS) or mastectomy when unfavorable prognostic factors are present 1, 2, 3, 4. Meta‐analyses by the Early Breast Cancer Collaborative Group showed that, compared with surgery alone, postoperative RT reduced the relative risk of ipsilateral recurrence and the breast cancer mortality rate, while improving disease‐free survival 5, 6.

Despite these advantages, treatment length was one of the main drawbacks of RT. In fact, RT takes 5–6 weeks (which lengthen to 6 or 7 weeks if boost is administered) as 1.8–2 Gy daily is delivered for 5 days per week up to a total dose of 50‐50.4 Gy, (60–70 Gy with boost) 2, 3, 7. Nowadays, a standard approach after BCS is a hypofractionated schedule which reduces treatment to 3–4 weeks and is associated with good outcomes. Hypofractionated RT schedules are not generally recommended after mastectomy as only two of three main randomized clinical trials included a few patients who had received mastectomy 2, 3. Another way to shorten RT in low‐risk breast cancer patients after BCS, is to irradiate only the initial tumor site with a safety margin round it, instead of the whole breast. Indeed, partial breast irradiation (PBI) as delivered by brachytherapy or IORT, takes only 1–4 days 2, 3.

Further barriers to RT access and appropriate treatment are age, comorbidity 6, 8, 9, 10 psychosocial and economic factors 11, 12, 13, 14, health service resources, and organization 7, 15, 16. Finally, and perhaps most important of all, residence at some distance from the RT center was reported to exert an improper impact on the delivery of appropriate RT (at least in large countries with sparsely populated areas)^17^, ^18^, ^19^, ^20^.

This study was designed to investigate factors influencing the probability of receiving appropriate RT in Umbria, Italy and to assess whether omission of RT affected survival. Umbria, a hilly region of 8.456 km² in central Italy with a population of 884,268 according to the 2011 Census, has two main towns and isolated rural areas. Like the other Italian regions, it has an autonomous health service which is free at point of care. Large university hospitals in Perugia (RT1) and Terni (RT2) have long provided RT facilities, while smaller RT centers in Città di Castello (RT3) and Spoleto (RT4) were opened in 2003 and 2007, respectively. A breast cancer screening program was started in 1998 for women in the 50–69 age group. Umbria has a high incidence of breast cancer (world‐standardized rate 84.3 in 2009–2013) and although it is the leading cause of cancer death in females (age standardized mortality rate: 13.9 in 2009–13), mortality is trending downward (1994–2013 annual percentage change −1.73*)^21^.

## Materials and Methods

### Data sources and quality

Inclusion criteria were residence in Umbria, age under 80 years old; primary infiltrating or in situ breast cancer as defined by the International Classification of Diseases, 10th Revision: C50‐50.9; D05‐D05.9 22; cancer diagnosis between 2001 and 2011; tumor stage <IV; breast cancer surgery in Umbria; indications to postoperative RT 2, 3, 4.

Cases were obtained from the Umbria Regional Cancer Registry 21. Data on breast cancer patients who had undergone RT were obtained from hospital discharge and/or outpatients records (67.7%) or from RT treatment archives in each of the four RT centers in Umbria (32%). Inter‐regional payment claims showed that 10 patients (0.3%) had undergone surgery in Umbrian hospitals and RT elsewhere.

### Ethics approval

Cancer registry data were handled according to Italian law and cancer registry regulations 23.

### Study cohort

A total of 8,368 women with breast cancer were registered in the study period. Excluded from this study were: (1) 36 patients with cause of death certification only (DCO); (2) 35 with malignant breast disease other than carcinoma (International Classification of disease for Oncology, third edition 24: codes 8800–9120); (3) 639 with stage IV disease; (4) 277 cases of previous cancer other than breast; (5)1871 patients for whom RT was not indicated (mastectomy with pT1‐pT2 pN < 2, according to Italian National Guidelines 2, 3); (6) 435 women ≥80 years old; and (7) 340 cases who received breast cancer surgery outside Umbria.

Of the 4735 women who were included in the present analyses, 4357 had been treated with BCS (3877 (81.9%) had early stage invasive breast cancer and 480 (10.1%) in situ breast cancer); 378 patients (8%) with pT3‐pT4, any pN or pT1‐pT2 pN≥2 had undergone mastectomy.

Thirty‐four patients receiving BCS which was converted to mastectomy were included in the mastectomy group.

### Study variables

The study period was subdivided as follows: 2001–2002, 2003–2007, and 2008–2011, in accordance with the number of active RT centers. Results are presented according to time period.

Variables included RT administration or not, age, socioeconomic status, comorbidity score, pT, pN, and type of surgery (BCS vs. mastectomy).

Comorbidities were derived from hospital discharge data. Hospital ICD‐9‐CM codes in the 5 years preceding breast cancer diagnosis were used to calculate the Deyo implementation of the Charlson score 25.

The Italian deprivation index (IDI), which is based on data from the Italian National Census, was used as indicator of socioeconomic status 26. Data from the 2001 Census were applied for patients diagnosed up to 2005 and data from the 2011 Census for patients registered between 2006 and 2011. The IDI score, calculated at census tract level (on average 123 residents), was then transformed into quintiles.

### Geographic data

The distance from each patient's residence to the nearest RT center (four in Umbria and five elsewhere near the Umbria border) was measured by a geographic information system (GIS)‐based calculation that was integrated into our information management system 21. Using Google Maps, we calculated the shortest journey time (min) and distance (km) from homes to RT centers where each patient was treated and from 15 hospitals where breast surgery had been performed in the cohort to RT centers. We hypothesized that people living close the centers receive the major benefit in terms of reduced spatial barriers. Then, we calculated journey times to new RT centers as if they were already present in the 2 years before they opened. Finally, we calculated the odds ratios of treatment, respectively, for people living close to the new centers before and after the start of activity and for people living elsewhere to disentangle the influence of increased availability and regional trend from spatial barriers. All distances were estimated according to municipality of residence, using multiple imputations 27 for 45 women (1%) whose addresses were missing.

### Statistical analysis

The chi‐square test assessed the impact of study variables on RT administration. *P* < 0.05 was considered significant. Logistic regression models were fitted to data to estimate odds of not receiving appropriate RT and to investigate the influence of journey times and distances on RT. Three hundred and twenty‐five patients receiving intra‐operative RT or brachytherapy were not included in these analyses.

The variables with *P* < 0.05 were included in the models applying a backward procedure. Validity of the models was checked using the Hosmer and Lemeshow goodness‐of‐fit test. Likelihood‐ratio test was used to evaluate statistical significance of each variable in the models. Log‐likelihoods of the full model and (Akaike Information Criterion) AIC*n was also reported for the final models.

Multiple imputations, with the chained equation method, were used to include data from 409 cases (9.3%) with at least one missing value. In accordance with White et al. (2011), analyses were based on 50 imputed datasets 28. The imputation models did not include vital status.

The propensity score 29 was calculated to compare survival in patients who received or did not receive RT as it eliminates or reduces confounding bias due to indication to treatment in nonrandomized cohort studies 30. Propensity scores for 4364 cases were matched one‐to‐one, that is, matching a patient treated with RT to the untreated patient with the closest propensity score without replacement. Rubin's tests and the sensitivity analysis assessed the results of propensity score matching 31, 32.

Overall survival was calculated in the propensity score‐matched groups using the Kaplan–Meier method. As recommended for Register data analysis 33, Pohar Perme's estimator 34 calculated net relative survival which provides a survival estimate that is analogous to disease‐specific survival.

A GLM Poisson approach was used to model excess mortality by treatment 35. All analyses were conducted using STATA 14.2 (Stata Corp ltd,TX) 36.

## Results

After either BCS or mastectomy, appropriate RT 2, 3, 4 was administered to 3962/4735 women (83.7%; 95% CI: 82.6–84.7%), increasing markedly over the study timeframe. Table 1 reports study variables that were significantly associated with RT omission in univariate analysis. All study variables had low missing levels. Omission of appropriate RT decreased from 27% in 2001–2002 to 10% in 2008–2011, while use of IORT/BRT increased from 0.2% to 9.6% in the same time frame. High percentage of RT omission was associated with pT4 (35%), in situ cancers (31%), mastectomy (27%), severe comorbidities (34%), age between 70 and 80 years old (29%), >40 min journey from surgery hospital to RT center (27%), and the 2001–2002 study time period (27%).

**Table 1:** Distribution of study variables by RT

Variables	RT NO		3D conformal RT		IORT‐BRT		TOTAL	*P*‐ value
	*N*	%	*n*	%	*n*	%	*N*	
Age at diagnosis (years)								<0.001
<40	27	14.5	159	85.0	1	0.5	187	
40–49	128	13.8	775	83.5	25	2.7	928	
50–59	155	13.1	966	81.9	59	5.0	1180	
60–69	159	11.4	1114	79.7	124	8.9	1397	
70–80	304	29.1	623	59.7	116	11.2	1043	
Comorbidity score								<0.001
0	641	14.9	3359	78.3	290	6.8	4290	
1–2	97	28.4	220	64.3	25	7.3	342	
>2	35	34.0	58	56.3	10	9.7	103	
Socioeconomic status								0.02
1	179	17.9	743	74.1	80	8.0	1002	
2	138	14.9	725	78.3	63	6.8	926	
3	179	19.3	680	73.3	69	7.4	928	
4	121	14.0	687	79.3	58	6.7	866	
5	140	15.6	708	78.7	51	5.7	899	
X	16	14.0	94	82.5	4	3.5	114	
T								<0.001
Is	148	30.7	316	65.8	16	3.3	480	
1	397	12.6	2472	78.7	273	8.7	3142	
2	142	16.5	687	80.0	30	3.5	859	
3	21	26.2	59	73.8	0	0.0	80	
4	38	35.2	65	60.2	5	4.6	108	
Unknown	27	40.9	38	57.6	1	1.5	66	
N								0.001
0	431	14.6	2247	75.7	289	9.7	2967	
1	120	12.5	814	84.9	25	2.6	959	
2	69	14.2	415	85.4	2	0.4	486	
Unknown	153	47.4	161	49.8	9	2.8	323	
Surgical procedure								0.001
Breast‐conserving surgery	673	15.4	3362	77.2	322	7.4	4357	
Mastectomy	100	26.4	275	72.8	3	0.8	378	
Period of diagnosis								0.001
2001–2002	235	27.3	624	72.5	2	0.2	861	
2003–2007	354	17.2	1540	75.4	148	7.2	2042	
2008–2011	184	10.0	1473	80.4	175	9.6	1832	
Journey time (min) from home to RT center								0.001
0–20	266	13.4	1577	79.2	147	7.4	1990	
21–40	305	17.4	1341	76.5	107	6.1	1753	
40+	196	20.7	680	71.8	71	7.5	947	
Unknown	6	13.3	39	86.7	0	0.0	45	
Journey time (min) from surgery hospital to RT center								0.001
0–20	284	11.7	1884	77.4	265	10.9	2433	
21–40	370	19.9	1437	77.3	53	2.8	1860	
>40	119	26.9	316	71.5	7	1.6	442	
Total	773	16.3	3637	76.8	325	6.9	4735	

Multivariate analyses confirmed these findings. Socioeconomic status, which had emerged with low significance in the univariate analysis, became nonsignificant (*P* = 0.15). Journey time from surgery hospitals to the nearest RT center impacted more on RT omission than journey times from residence (Table 2).

**Table 2:** Logistic regression analysis with factors associated with not receiving RT: MODEL 1 ‐ distance from home to nearest RT; MODEL 2 ‐ distance from surgery hospital to nearest RT center

Variables	MODEL 1			MODEL 2		
	OR	95% CI	*P*‐value	OR	95% CI	*P*‐value
Age						
<40 (ref)	1			1		
40–49	0.95	(0.60–1.51)	0.83	0.96	(0.60–1.52)	0.86
50–59	0.88	(0.55–1.38)	0.58	0.88	(0.55–1.39)	0.58
60–69	0.74	(0.47–1.17)	0.2	0.75	(0.47–1.18)	0.22
70–80	2.69	(1.71–4.22)	<0.001	2.7	(1.72–4.24)	<0.001
Year of diagnosis						
<2003	3.47	(2.75–4.39)	<0.001	3.37	(2.66–4.26)	<0.001
2003–2007	2.02	(1.65–2.49)	<0.001	2.07	(1.69–2.54)	<0.001
2008–2011 (ref)	1			1		
Comorbidities						
0 (ref)	1			1		
1–2	1.79	(1.35–2.36)	<0.001	1.82	(1.38–2.40)	<0.001
>2	2.55	(1.58–4.1)	<0.001	2.65	(1.65–4.26)	<0.001
Journey time RT–home (min)				–	–	–
0–20 (ref)	1			–	–	–
21–40	1.26	(1.04–1.53)	0.02	–	–	–
>40	1.47	(1.18–1.84)	0.001	–	–	–
Journey time RT–surgery(min)	–	–	–			
0–20 (ref)	–	–	–	1		
21–40	–	–	–	1.55	(1.29–1.86)	<0.001
>40	–	–	–	2.36	(1.81–3.08)	<0.001
Surgical procedure						
Conservative (ref)	1			1		
Mastectomy	2.01	(1.30–3.09)	0.002	2.00	(1.29–3.08)	0.002
T						
Is	2.28	(1.27–4.10)	0.006	2.38	(1.32–4.29)	0.004
1	0.62	(0.35–1.07)	0.08	0.63	(0.36–1.09)	0.1
2	0.71	(0.41–1.22)	0.21	0.73	(0.42–1.26)	0.26
3	1.05	(0.52–2.09)	0.89	1.08	(0.54–2.16)	0.83
4	1			1		
N						
0	1.82	(1.23–2.70)	0.003	1.83	(1.23–2.71)	0.003
1	1.22	(0.82–1.82)	0.33	1.23	(0.82–1.84)	0.31
2	1			1		
*Log‐likelihoods full model	−1514.08			−1497.667		
AIC*n	3078.16			3045.33		

**Figure 1:**
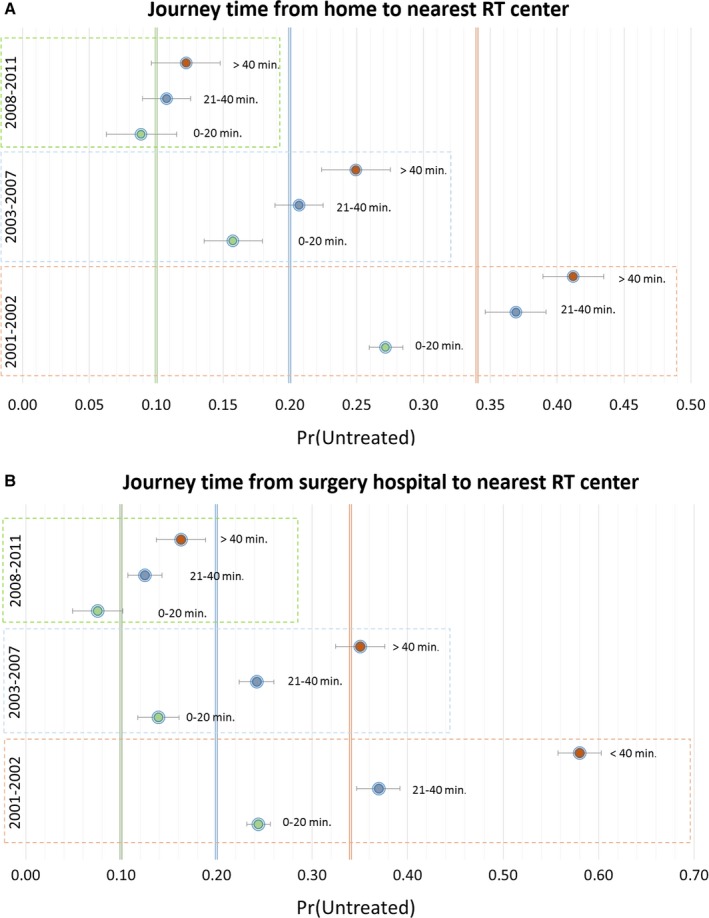
Adjusted probability of RT omission by period and distance based on logistic regression models. (A) Journey time from home to nearest RT. (B) Journey time from surgery hospital to nearest RT center. RT, Radiotherapy.

Figure 1 illustrates the adjusted probability of RT omission, over the study timeframe. The high probability of RT omission was markedly impacted by distance from home or surgery hospital to RT center in 2001–2002 and dropped significantly by 2008–2011.

**Figure 2:**
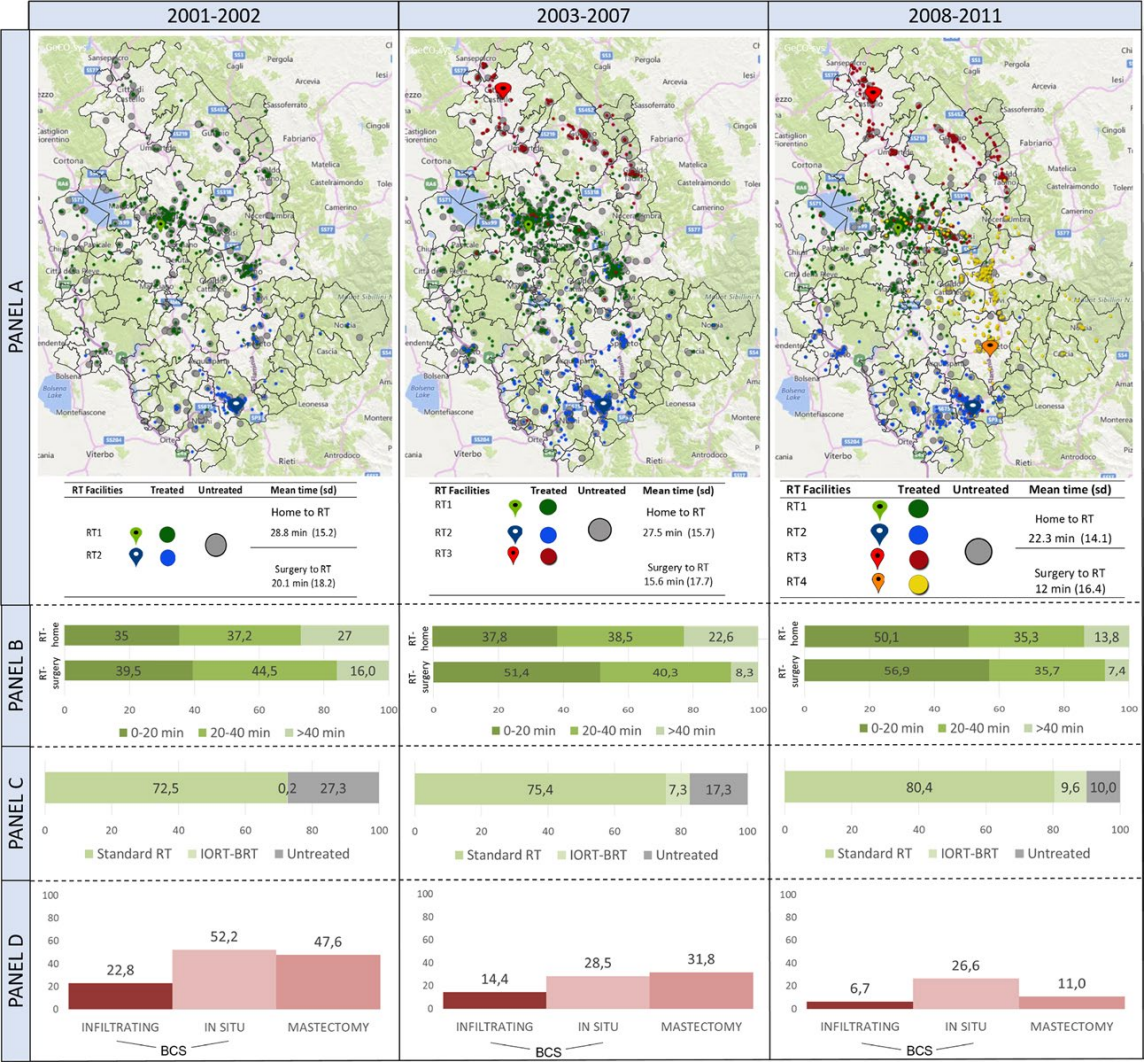
RT treatment by period. Panel (A): distribution of study cases and RT facilities (colored marks). Colored points indicates centers where RT was performed. Gray points correspond to untreated patients. Panel (B) distribution of study cases by distance, RT‐home and RT‐surgery (min). Panel (C) distribution of patients by RT treatment. Panel (D) Percentage of untreated patients by surgery type and disease behavior. RT, Radiotherapy.

Figure 2 maps distribution of patients in relation to the RT centers they attended, showing more patients were treated as the number of RT centers increased from two to four over time. The new RT facilities shortened mean journey times for patients from 29 to 22 min. From 2001 to 2002, 27% patients were living more than 40 min journey from the nearest RT center compared with only 14% in 2008 to 2011. Journeys >40 min from surgery hospital to RT centers dropped from 16% to 7%. RT omission dropped sharply from 48% in 2001–2002 to 11% in 2008–2011 after mastectomy for high‐risk tumors and from 52% to 28% in 2007–08, remaining stable from 2003 afterward at 27% for in situ cancers.

Trend toward administration of appropriate RT was more marked in patients living near the new RT centers. The analysis comparing the first 2 years of the new RT centers' activity versus 2 years before their opening, showed that the OR of RT omission was 0.64 (95% CI: 0.48–0.85) for patients living in the catchment area of the new centers versus 0.74 (95% CI: 0.61–0.88) for patients living elsewhere in Umbria.

**Figure 3:**
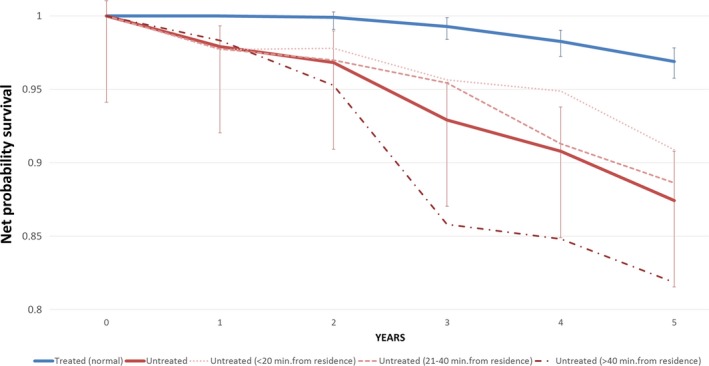
Net probability survival of propensity score‐matched breast cancer patients by RT and, among untreated women, by travel time to the nearest RT facility. RT, Radiotherapy.

Survival data were high quality as no case was lost to follow‐up and the percentage of Death Certificate Only cases was 0.4%. Median follow‐up was 8.1 years with the last census on 31 December 2015. In propensity score‐matched groups, 5‐year overall survival rates were 0.94 (95% CI: 0.93–0.95) for patients who received RT and 0.82 (95% CI: 0.79–0.85) for untreated patients. The 5‐year net survival probabilities were 0.97 (95% CI: 0.96–0.98) for treated versus 0.87 (95% CI: 0.83–0.91) for untreated patients (Fig. 3). Excess mortality for untreated patients was significantly higher (4.58; 95% CI: 2.99–7.01).

For untreated patients residing at >40 min journey time from the nearest RT center, the 5‐year net survival was 0.82 (95% CI: 0.73–0.88) versus 0.91 (95% CI: 0.83–0.95) in untreated patients living <20 min journey time away (Fig. 3). Compared with propensity score‐matched treated patients, excess mortality in the untreated was, respectively, 7.90 (95% CI: 3.31–18.87) and 3.22 (95% CI: 1.65–6.29). The 5‐year net survival in treated patients did not vary with journey time from RT center (data not shown).

## Discussion and Conclusions

The present investigation is, as far as we know, the first study to illustrate the impact of spatial barriers upon delivery of appropriate postoperative RT for breast cancer patients in Europe. In fact, several reports from North America and Australia and a recent meta‐analysis provided evidence of increasing risk of undertreatment with distance from RT facilities 37, 38, 39, 40. In British Columbia, Liu et al. 41 found that travel times of >2 h influenced RT delivery for breast cancer patients.

Focussing on an Italian region with a small population spread over rural areas and using high quality population‐based data, this study showed that journey times of over 40 min hindered appropriate RT for breast cancer patients, confirming other findings 38, 42. Despite large differences in population size and area, our results are in line with findings from a Piedmont study which analyzed overall utilization of RT for all cancer patients 20.

Journey time between the surgery hospital and the nearest RT center impacted more on appropriate RT delivery than travel time from residence. Interpreting this finding is arduous but hypotheses may be patient‐ or health service‐related. They include patient educational level, self‐selection of patients seeking care in hospitals near residence, surgeon decisions, lack of referrals from remote surgical hospitals due to lack of a multidisciplinary oncology team or consultants. One limitation of this study is inability to assess the impact of unmeasured and residual confounders even though propensity scores were used to match treated and untreated patients, and another is lack of information on multidisciplinary care and preoperative consultation with a radiation oncologist and diagnosis at screening. In fact, an American study reported that preoperative consultation with a radiation oncologist was linked to high probability of RT delivery 18.

Present analyses clearly demonstrated that survival was poorer in our RT candidates who were left untreated, confirming meta‐analysis findings that RT omission worsens outcomes 5, 43. Dragun et al. who also reported worst outcomes for patients who resided at some distance from RT centers, suggested that, besides RT omission 44, missing aspects of multidisciplinary care might impact upon survival rates in early breast cancer. In this study, long journey times were associated with poor survival only in untreated patients who may well have missed out on some, if not most, aspects of multidisciplinary care.

Unlike other studies 13, 14, RT use in Umbria was not associated with socioeconomic status; perhaps, because there are no great inequality gaps among residents in Umbria and, as all over Italy, the Regional Health Service of Umbria is free at point of use; so, there is no financial burden for patients beyond transport costs.

Evidence of inappropriate care and poorer outcomes led to the establishment of new RT centers in Umbria, as in small rural communities in Canada 45.Concurring with Liu et al. 41, this study provided evidence that new RT centers contributed to reduce the number of untreated patients which fell from more than 1 in 4 in 2001–2002 to 1 in 10 in 2008–2011. This drop was more marked for patients living near a new RT center, showing shorter journey times impacted more than increased RT availability 46. Consequently, the higher than average number of RT centers in Umbria (4.6 per million of inhabitants vs. 3 Italian average) 47, led to low levels of inappropriate treatment. One might reasonably object that doubling RT facilities was a costly solution to RT underuse, and that the Umbria model could not serve as a solution to RT accessibility in all European regions with rural areas, considering that the trend in modern medicine is toward large all‐inclusive centers for cancer treatments. In general, the number of RT centers that are set up to reduce the influence of spatial barriers needs to be balanced against costs and loss of quality which was not, however, observed in small RT facilities but was, for example, found in surgical units 48, 49. Quality of care should undoubtedly be monitored. In our view, an optimal solution is the interactive hub and spoke model, ensuring appropriate care for all patients by means of the oncology network.

In conclusion, for breast cancer patients, journey times emerged as a major determinant of RT delivery in the Italian region of Umbria. RT omission was associated with worst health outcomes, particularly among patients who resided in rural areas and underwent surgery at nearby hospitals. Over the 11‐year study period, the Umbria Regional Health Service established two new RT centers which contributed to its present satisfactory delivery of appropriate RT.

## Conflicts of Interest

The authors declare that they have no competing interests.
